# An Efficient Automatic Fruit-360 Image Identification and Recognition Using a Novel Modified Cascaded-ANFIS Algorithm

**DOI:** 10.3390/s22124401

**Published:** 2022-06-10

**Authors:** Namal Rathnayake, Upaka Rathnayake, Tuan Linh Dang, Yukinobu Hoshino

**Affiliations:** 1 School of Systems Engineering, Kochi University of Technology, 185 Miyanokuchi, Tosayamada, Kami 782-8502, Kochi, Japan; hoshino.yukinobu@kochi-tech.ac.jp; 2 Department of Civil Engineering, Faculty of Engineering, Sri Lanka Institute of Information Technology, Malabe 10115, Sri Lanka; upaka.r@sliit.lk or; 3 School of Information and Communications Technology, Hanoi University of Science and Technology, No. 1, Dai Co Viet Road, Hanoi 100000, Vietnam; linhdt@soict.hust.edu.vn or

**Keywords:** automated image classification, cascaded-ANFIS, confusion matrix, features descriptors, Fruit-360 dataset

## Abstract

Automated fruit identification is always challenging due to its complex nature. Usually, the fruit types and sub-types are location-dependent; thus, manual fruit categorization is also still a challenging problem. Literature showcases several recent studies incorporating the Convolutional Neural Network-based algorithms (VGG16, Inception V3, MobileNet, and ResNet18) to classify the Fruit-360 dataset. However, none of them are comprehensive and have not been utilized for the total 131 fruit classes. In addition, the computational efficiency was not the best in these models. A novel, robust but comprehensive study is presented here in identifying and predicting the whole Fruit-360 dataset, including 131 fruit classes with 90,483 sample images. An algorithm based on the Cascaded Adaptive Network-based Fuzzy Inference System (Cascaded-ANFIS) was effectively utilized to achieve the research gap. Color Structure, Region Shape, Edge Histogram, Column Layout, Gray-Level Co-Occurrence Matrix, Scale-Invariant Feature Transform, Speeded Up Robust Features, Histogram of Oriented Gradients, and Oriented FAST and rotated BRIEF features are used in this study as the features descriptors in identifying fruit images. The algorithm was validated using two methods: iterations and confusion matrix. The results showcase that the proposed method gives a relative accuracy of 98.36%. The Fruit-360 dataset is unbalanced; therefore, the weighted precision, recall, and FScore were calculated as 0.9843, 0.9841, and 0.9840, respectively. In addition, the developed system was tested and compared against the literature-found state-of-the-art algorithms for the purpose. Comparison studies present the acceptability of the newly developed algorithm handling the whole Fruit-360 dataset and achieving high computational efficiency.

## 1. Introduction

Given the tremendous growth of the current population rate, the foods that we consume are a significant concern. Fruits are an essential consuming food in the day-to-day life pattern of most people and a highly recommended source of nutrient supply by nutritionists. Various strategies for fruit detection utilizing computer vision technologies have been used for many years. These approaches are used to categorize and distinguish various types of fruits from a collection of photographs. Fruit categorization is still regarded as a contentious and complex problem not only in the research world but also in the practicing industries. Identifying the class of a particular fruit, for example, allows grocery staff to quickly calculate its price [1]. Furthermore, nutritional recommendations are beneficial in assisting consumers in picking appropriate food varieties that satisfy their nutrient and well-being demands [2,3]. Fruit categorization techniques are frequently employed in most food facilities for automated packing.

The fruit types and sub-types are location-dependent (varies from location to location even in the same country), thus manual fruit categorization is still a challenging problem. This vast disparity is centered on the availability of population-dependent and region-dependent fruits, as well as the required elements in the fruits [3]. Artificial Intelligence (AI) and Machine Learning (ML) approaches are utilized in various applications to give optimal solutions to challenges faced in a variety of disciplines such as image analysis, speech recognition, forecasting, prediction, massive dataset analysis, and marketing [4]. Thus, the rapid advancement in computer vision and machine learning, particularly in the recent decade, has drawn the attention of various researchers to the use of established approaches in automatic fruit categorization. Researchers frequently employed elements linked to exterior quality descriptors in their study, such as form, size, texture, and color [5,6]. In general, most of the suggested classifiers were either constrained to a certain kind of fruit or showcased poor accuracy. Many of the classification systems are purely based on Neural Network (NN) algorithms and very few approaches in the literature were based on Fuzzy Logic (FL).

## 2. Related Works

Several automatic fruit and vegetable categorization algorithms have been introduced in recent years by experts. VeggieVision [7] was the first product from a significant attempt in recognizing the vegetables and fruits. This device had an integrated scale as well as a digital camera. When an item was placed on the scale, the camera captures the image. Color, texture, and other characteristics were retrieved and compared to previously stored characteristics of distinct product varieties. These stored characteristics were acquired throughout the training procedure. When the training and testing datasets were from the same store, the best pick had a classification accuracy of 82.6%. The classification accuracy decreased dramatically when the training and testing datasets were from separate stores.

Seng and Mirisaee [8] suggested another fruit detection method based on color, shape, and size. The color was represented by the mean RGB value, shape by the measure of roundness, and size by the area and perimeter values. These feature values were then used to classify data using the k-nearest neighbor technique. Despite the excellent accuracy rates reported, the training and testing datasets were relatively small.

Two different machine learning-based fruit categorization algorithms are proposed by Wang et al. [9]. Wavelet entropy, principal component analysis, feed-forward neural networks trained with Fitness-Scaled Chaotic Artificial Bee Colony (FSCABC), and bio-geography-based optimization techniques were used in their procedures. The categorization accuracy for both approaches was 89.5%, which is higher compared to the earlier approaches.

However, Pennington and Fisher [3] were the first scientists to utilize the clustering approach to categorize fruits and vegetables in 2009. They have employed a dataset having 104 common fruits and vegetables for classification. Visible spectroscopy was used by Pholpho et al. [10] to distinguish damaged and undamaged fruits. Furthermore, Yang et al. [11] presented an estimating approach for blueberry fruit identification using multi-spectral image analysis. In contrast, computer vision and multi-class Support Vector Machine (SVM) were used to categorize distinct varieties of fruit with an 88.20% accuracy [12]. Later, eight different citrus fruits were identified using Raman spectroscopy as a quick and non-destructive measure using two analytic approaches (hierarchical cluster and principal component) [5]. In addition, Fadhel et al. [13] employed color segmentation to identify immature strawberries. They have used two different methods for classification: color thresholding and K-means clustering. The results indicate that the color thresholding results outperform the clustering method. The literature also presents the related studies in impurity identification in olive oil using similar techniques of computer vision and machine learning [6]. Furthermore, Breijo et al. [14] used an electronic nose (also known as a piece of olfactory sampling equipment) to characterize the odor of Diospyros kaki (Persimmons). The system’s operating parameters have the power to impact the changeable configurations, allowing the system to be flexible.

On the other hand, Fan et al. [15] used an artificial neural network with two hidden layers to predict the texture features derived from a food-surface picture. The back-propagation method was utilized for training the neural network. However, the neural network approach had some disadvantages, including behaving as a black box, intensive duration of development, and the requirement of a lot of data.

However, Omid et al. [16] presented an expert system for extracting size and then defecting information using machine vision and Fuzzy Logic. This approach employed two different types of membership functions, including triangular and trapezoidal. In addition, the study was evaluated using the Correct Classification Rate (CCR), and overall accuracy of 95.40% was obtained.

Another automatic fruit categorization system was proposed based on the fitness-scaled chaotic artificial bee colony algorithm [17]. The authors have compared the performance of the proposed algorithm with three well-known AI methods. However, the proposed FSCABC-FNN method has only shown an 89.10% accuracy outperforming other algorithms. In addition, Khanmohammadi et al. [18] have proposed a classification method based on Near-Infrared Spectrometry (FT-NIR) and Square SVM. They have succeeded in obtaining a meager prediction error rate of 2%. Furthermore, a texture-based method that involves descriptor computation and interest-point feature extraction was proposed [19]. They stated that the study shows excellent results on a single image detection rate having 85.00% and 100.00% for pineapple and bitter lemon fruits, respectively. Date fruits were identified using Weber’s local descriptor and local binary pattern approaches and SVM for classifier and Fisher discrimination ratio for feature selection [20]. This study has considered three feature descriptors such as color, texture, and shape. The proposed algorithm shows a 98.00% accuracy after the dimension reduction using Fisher Discrimination Ratio (FDR).

The literature presents many related research studies based on Convolutional Neural Networks (CNN) to the Fruit-360 dataset in recognizing the fruits. A CNN-based VGG16 model used developed by Siddiqi [21] to classify 72 classes of the Fruit-360 dataset and the author has obtained 99.27% accuracy in total. Ghazanfar et al. [22] have presented a model using Deep Convolutional Neural Networks (DCNN) to classify the same dataset (Fruit-360) and acquired a 92.00% recognition rate. The individual classes of the Fruit-360 dataset were combined to create new classes in this research. Therefore, the total number of categories was reduced to 16 for the classification. This created the problem of robustness. In addition, Ghosh et al. [23] have introduced an image classification model using ShufleNet V2 that is based on the CNN algorithm. They have obtained an accuracy of 96.24% for 40 classes in the Fruit-360 dataset. Furthermore, Postalcıoğlu [24] has also presented a model based on CNN. Three different optimizers including Stochastic Gradient Descent with Momentum (SDGM), Adaptive Moment Estimation (Adam), and Root Mean Square Propagation (RMSPROP) were used in that analysis to evaluate the results. The results were 98.08%, 98.83%, and 99.02% accurate, respectively. However, the research was only conducted for 48 classes in the Fruit-360 dataset. Therefore, the study was not a comprehensive work. In another study, Ziliang et al. [25] have showcased an accuracy of 98.06% for a classification model using the CNN algorithm. However, they have extended the analysis for 81 classes of the Fruit-360 dataset.

A deep review of related literature presents the following drawbacks and shortcomings.

1. The studies required expensive sensors such as weight, dew, heat, chemical, gas-sensitive, and infrared light to model the classification.
2. The classifiers are only capable of recognizing a few types of fruits, not the whole Fruit-360 dataset.
3. The system performance is insufficient, owing primarily to closely related texture, color, and shape properties.
4. The classification precision falls short of the standards for typical applications.
5. The algorithms required a higher computational power.

Therefore, this research study proposes a new algorithm based on the Cascaded Adaptive Neuro Fuzzy Inference System (Cascaded-ANFIS) [26] to fill the above-identified research gaps in the literature and then to present a much enhanced and robust model to identify the fruits based on their properties. The major contributions of the presented research can be listed as follows.

1. This study proposes a novel structure for the Cascaded-ANFIS algorithm for image classification.
2. The system is designed using nine state-of-the-art feature descriptors (including Color Structure (CS), Region Shape (RS), Edge Histogram (EH), Column Layout (CL), Gray-Level Co-Occurrence Matrix (GLCM), Scale-Invariant Feature Transform (SIFT), Speeded Up Robust Features (SURF), Histogram of Oriented Gradients (HOG), and Oriented FAST and rotated BRIEF features (ORB)).
3. The total dataset of 131 classes is used for the classification.
4. The novel system can reduce the dimension input to different features due to the usage of the feature reduction method.
5. Comparison of the accuracy with the state-of-the-art algorithms (including CNN with Stochastic Gradient Descent with Momentum, CNN with Adaptive Moment Estimation, CNN with RMS propagation, Customized Inception V3, Customized VGG 16, Customized MobileNet, Vanilla MobileNet, ShufeNet V2, DCNN, and ResNet18).
6. The comparative computational power is relatively inexpensive while providing an accuracy up to 98.36%.

## 3. Proposed Methodology

### 3.1. The Fruit-360 Dataset

Fruit-360 is a dataset which has 90,483 fruit photos (67,692 in the training set and 22,688 in the test set) [27]. The collection contains 131 different varieties of fruits, and each fruit has an image only capturing one fruit. These images are 100 × 100 pixels in size. The training set and test set for each fruit type contain a somewhat different number of photos, although, in most situations, roughly 70% training images and 30% test images are provided for each fruit type. These images are obtained by filming a brief video of fruit for twenty seconds while it is slowly spun by a motor and then extracting frames/images from that movie. A white sheet of paper is used as the background for the capture. The background of each fruit is then eliminated by a specific algorithm. The varying light intensity can impact the background; therefore, it has to be removed.

### 3.2. The Cascaded-ANFIS Algorithm

The development of the classifier requires a combination of several theories. The core algorithm used in this study is the Cascaded-ANFIS algorithm and a brief introduction is presented in the following subsection.

ANFIS combines two different algorithms, such as NN and FL. Therefore, ANFIS showcases the advantages of both NN and FL algorithms [26]. ANFIS has six layers in its structure. Usually, the first layer is the input while the last layer is the output. The membership functions are generated in the 2nd layer using FL while the cumulative product of these membership functions is generated in the 3rd layer. The 4th layer normalizes the output from the 3rd layer while the 5th layer is used to defuzzify the previous outputs to generate the final value. Equations (1)–(5) present the corresponding calculations for each level in general ANFIS algorithm.

(1) $$\mu_{Ai}=max\left(min\left(\frac{x-a_{i}}{b_{i}-a_{i}},\frac{c_{i}-x}{c_{i}-b_{i}}\right),0\right),i=1,2$$

(2) $$O_{2,j}=w_{j}=\mu_{Ai}\left(x_{1}\right)*\mu_{Bi}\left(x_{2}\right)$$

(3) $$O_{3,j}=w_{j}=\frac{w_{j}}{\sum w_{i}},j=1,2,...,n$$

(4) $$O_{4,j}=w_{j}f_{j}=w_{j}\left(p_{j}x_{1}+q_{j}x_{2}+r_{j}\right)$$

(5) $$O_{5,1}=\sum_{j=1}^{n}w_{j}f_{j}$$

where $\mu_{Ai}$ is the *A*th membership function for the input i=1,2 and x,a,b, and *c* are input values and the premise parameters in the triangular membership function, respectively. $O_{\left(k,j\right)}$ is the output of respective layer k and *j*th rule. $w_{j}$ is the firing strength of the *j*th rule and p,q, and *r* are the consequent parameters for the defuzzification.

**Figure 1:**
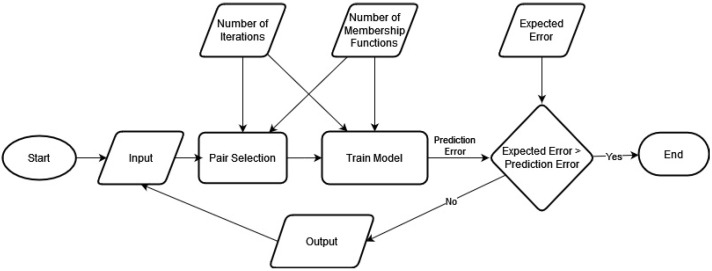
Flowchart of the original Cascaded-ANFIS algorithm structure.

The ANFIS algorithm is repetitively used with two inputs, and one output in the Cascaded-ANFIS algorithm. The construction of the Cascaded-ANFIS algorithm is presented in Figure 1.

The Cascaded-ANFIS algorithm is made up of two major parts: the pair selection method and the training method. More information and technical details about Cascaded-ANFIS can be found in Rathnayake et al. [26,28]. ANFIS poses a major disadvantage when it is used with higher dimensional data. The computational complexity of the ANFIS algorithm mainly depends on the number of input features used in the system. Therefore, in related research, dimension reduction methods are generally used to overcome this effect. However, the computational complexity is easily handled by the innovative Cascaded-ANFIS algorithm. In addition, noisy data sets can also be handled by the unique techniques developed in Cascaded-ANFIS. However, this study is based on image data classification which provides an extensively large number of input dimensions to the system. Therefore, a state-of-the-art dimension reduction method was investigated to apply to solve this issue. Hence, three well-known Dimension Reduction (DR) methods were considered: Independent Component Analysis (ICA) [29], Principle Component Analysis (PCA) [30,31], and Multi-Dimensional Scaling (MDS) [32].

The results of using these DR methods are illustrated in the implementation of the algorithm section. A simple experiment was carried out to identify the best algorithm for dimension reduction. Three well-known datasets (vehicles by Siebert [33], breast cancers by Wolberg and Mangasarian [34], Musk 1 by Dietterich et al. [35]) were used to reduce the dimension using the Cascaded-ANFIS algorithm using all three methods. These datasets were selected based on different perspectives, such as field of interest and the number of inputs and outputs.

### 3.3. Image Data Analysis—Feature Extraction

Features are the key ingredient in implementing a classifier. Therefore, according to the literature, nine feature descriptors are used to extract different features from the Fruit-360 image dataset. This section provides a brief introduction to each of these feature extraction methods. The first method is the Color Structure descriptor. It is based on histogram equalization, but it seeks and gives a complete description by differentiating localized color variations for each color [36]. The next feature descriptor is the Region Shape. The shape characteristics are less developed than their color and texture equivalents because of the intrinsic difficulties of portraying forms [37].

However, due to the variety of possible projections of a 3D object into 2D shapes, the complexity of each object shape, the presence of shadows, occlusions, non-uniform illumination, and varying surface reflectivity, it is not accessible to precisely segment an image into meaningful regions using low-level features. Therefore, the Column Layout feature descriptor was used as the third feature extraction method.

The Edge Histogram (EDH) descriptor represents the geometry of an image and is meant to depict the distribution of local edges inside pictures [38]. Therefore, the EDH descriptor was used as the fourth feature extraction method in this research. Edges are an essential attribute for viewing image information, and the histogram was used to characterize them. The homogeneous color histogram and texture feature cannot reproduce an image’s EDH-described qualities [39,40]. The fifth feature descriptor is the Gray Level Co-Occurrence Matrix (GLCM). It determines how frequently unique combinations of grey levels co-occur in an image or section of an image given an image made up of pixels, each with an intensity (a specific grey level). The GLCM contents are utilized in texture feature calculations to measure the change in intensity (also known as image texture) at the pixel of interest [41].

The sixth and seventh descriptors are Scale Invariant Feature Transform (SIFT) [42] and Speeded Up Robust Features (SuRF) [43]. SIFT characteristics include scale and rotation invariance and they have various advantages, including localization, distinctiveness, quantity, efficiency, and flexibility. On the other hand, SURF is a quick and trustworthy approach for encoding and estimating pictures in a local, similarity invariant way. The SuRF technique’s main appeal is its ability to calculate operators fast using box filters, enabling real-time tracking and object recognition applications.

The Histogram of Oriented Gradients (HOG) feature descriptor is the eighth feature extraction method used in this study. It is related to the Canny Edge Detector and the SIFT, and it is used in image processing to detect objects [44]. The method counts how many times a gradient orientation appears in a specific picture section. The ninth and the last feature descriptor was presented by Ethan et al. and it is called the Oriented FAST and Rotated BRIEF (ORB) [45]. The FAST key-point detector serves as the foundation for the ORB descriptor. ORB performs feature identification similarly to SIFT and SURF while being roughly two orders of magnitude faster. Because of its significant contributions, the ORB descriptor is employed as the feature extractor in many machine learning models [46,47].

### 3.4. Application Methodology—Novel Modified Structure for the Cascaded-ANFIS

**Figure 2:**
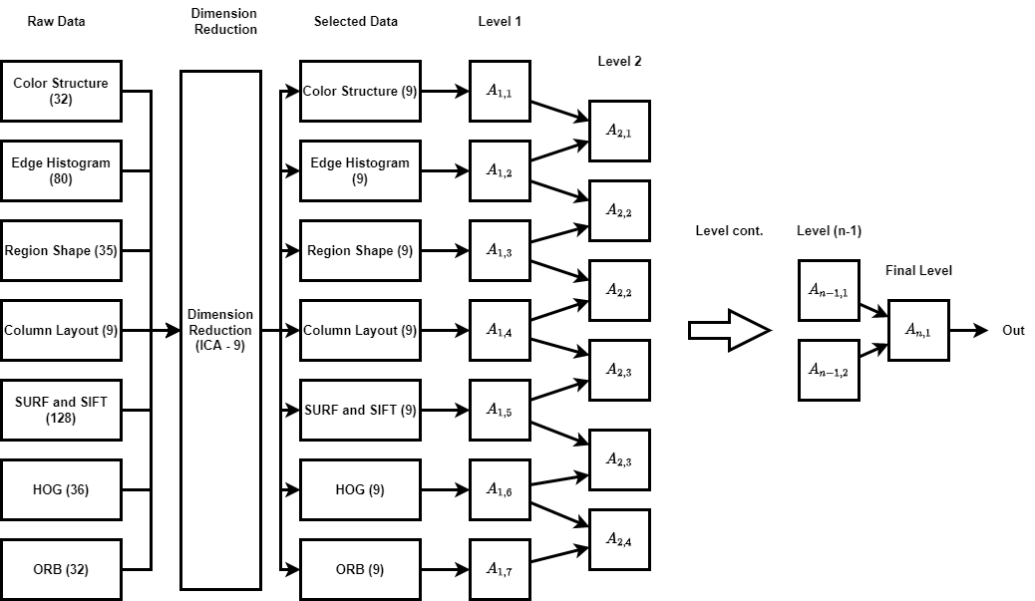
The Proposed Modified Novel Structure of Cascaded-ANFIS algorithm.

The flowchart for the developed Cascased-ANFIS algorithm is presented in Figure 2. In Figure 2, $A_{\left(i,j\right)}$ represents the ANFIS structure and *i* and *j* are the number of the levels and the number of the ANFIS structures in the corresponding level. Hence, there are seven ANFIS structures in the first level and represented as $A_{1,1}...A_{1,7}$.

A modified Cascaded-ANFIS algorithm had to be built to extract more features from a few descriptors from a single image (for example 352 features can be extracted as shown in Figure 2). However, reducing these features to a lesser number of meaningful features is challenging.

One of the main aspirations of this study is to generate a real-time system with higher accuracy compared to the existing algorithms. Therefore, reducing as many input dimensions as possible gives an added advantage in reducing time consumption and computational complexity. The reduction of input features was carried out considering each feature descriptor individually.

As shown in Figure 2, ICA was used as the feature reduction method in the developed model. The feature reduction was carried out for each set individually. The resulting feature set is the “Selected Data” (refer to Figure 2) and each set of features contains 9 features. The feature number was reduced to 63 (from 352) by using ICA. Then, the initial level of the modified Cascaded-ANFIS algorithm was started. The initial level of this structure uses seven inputs (consisting of 9 features) even though the usual Cascaded-ANFIS algorithm uses 2 inputs. Therefore, this modified architecture has seven Cascaded-ANFIS levels. Each ANFIS uses the previous output as the input to the current module.

### 3.5. Performance Analysis Techniques

The performance of the developed model was analyzed using a confusion matrix. A confusion matrix gives information about the predictions. Other classification matrices shown in Equations (6)–(12) were tested to understand the confusion matrix.

(6) $$Accuracy_{Avg}=\frac{\sum_{i=1}^{l}\frac{tp_{i}+tp_{i}}{tp_{i}+fn_{i}+fp_{i}+tn_{i}}}{l}$$

(7) $$Precision_{\mu }=\frac{\sum_{i=1}^{l}tp_{i}}{\sum_{i=1}^{l}\left(tp_{i}+fp_{i}\right)}$$

(8) $$Recall_{\mu }=\frac{\sum_{i=1}^{l}tp_{i}}{\sum_{i=1}^{l}\left(tp_{i}+fn_{i}\right)}$$

(9) $$FScore_{\mu }=\frac{\left(\beta^{2}+1\right)Precision_{\mu }Recall_{\mu }}{\beta^{2}Precision_{\mu }+Recall_{\mu }}$$

(10) $$Precision_{M}=\frac{\sum_{i=1}^{l}\frac{tp_{i}}{tp_{i}+fp_{i}}}{l}$$

(11) $$Recall_{M}=\frac{\sum_{i=1}^{l}\frac{tp_{i}}{tp_{i}+fn_{i}}}{l}$$

(12) $$FScore_{M}=\frac{\left(\beta^{2}+1\right)Precision_{M}Recall_{M}}{\beta^{2}Precision_{M}+Recall_{M}}$$

where $tp_{i},tn_{i},fp_{i},$ and $fn_{i}$ are True Positive, True Negative, False Positive, and False Negative respectively. In addition, *l* is the total number of classes and μ and *M* are the micro and micro-averaging. Each of these parameters conveys valuable information about the performance of the classification when the problem is multiclass [48]. The performance of the novel Cascaded-ANFIS model was tested and presented for its accuracy.

## 4. Results and Discussion

### 4.1. Feature Dimension Reduction

**Figure 3:**
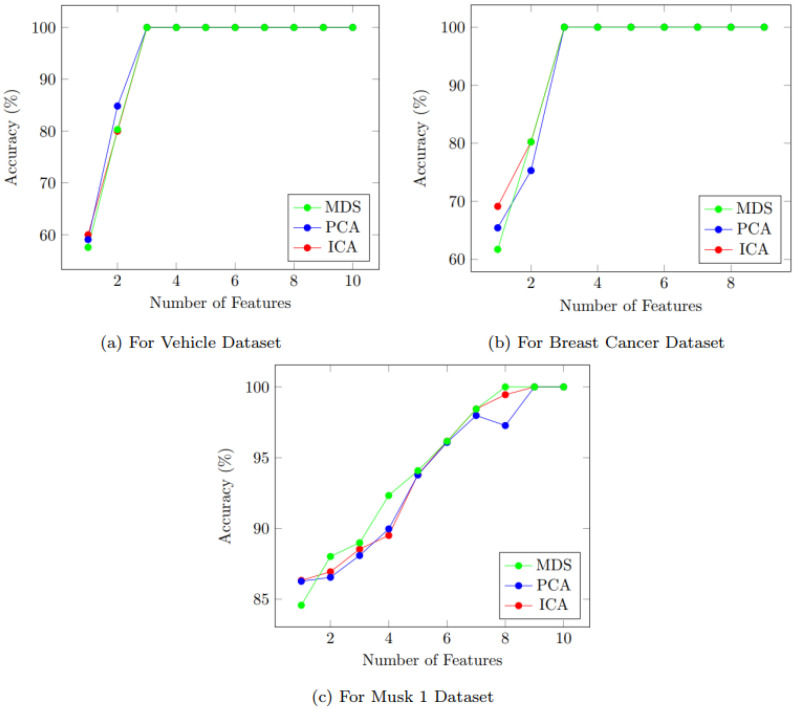
Accuracy comparison of feature dimension reduction algorithms when used on well-known datasets (breast cancer, vehicle, and Musk 1).

Figure 3a–c show the results of dimension reduction for three algorithms. They all reach very good accuracies and showcase similar variations. Therefore, the time consumption to perform these algorithms was considered a selection criterion.

**Figure 4:**
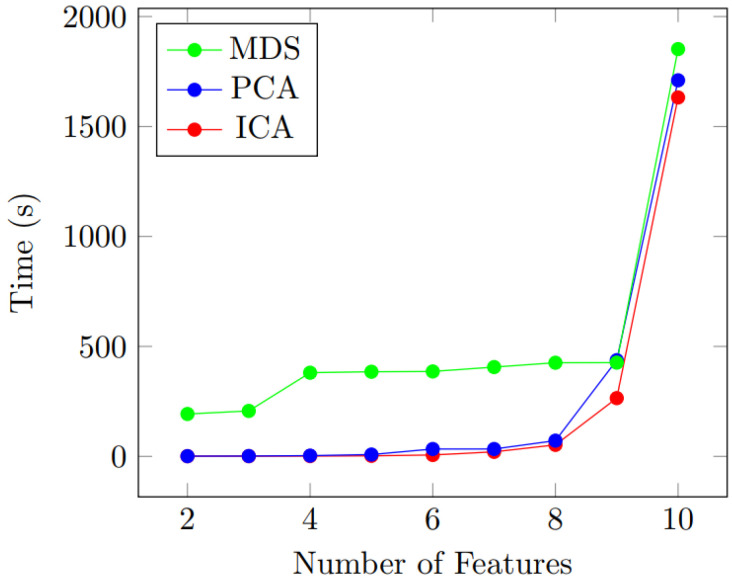
Time consumption for feature dimension reduction (time is denoted in seconds (s)).

Figure 4 presents the time consumption to perform the DRs. As it can be clearly seen, the time consumption is almost similar in ICA and PCA from features 2 to 10. However, MDS shows a longer calculation time when compared with the other two methods. For example, 0.99 s, 1.07 s, and 192.76 s were consumed at feature number two for ICA, PCA, and MDS, respectively.

However, with the increase in feature numbers, ICA and PCA have caught up with MDS. This can be seen at the feature number of 10. Nevertheless, ICA still shows better performance with respect to time consumption. At feature number 10, the time consumption is recorded as 1632 s, 1710 s, and 1852 s, respectively for ICA, PCA, and MDS. Therefore, the modified Cascaded-ANFIS algorithm was constructed using the ICA feature dimension reduction method.

### 4.2. Learning Behaviour by Iterations

The learning behavior performance of selected algorithms was compared with the novel Cascaded-ANFIS algorithm performances. A summary of the experiment is shown in Table 1.

The traditional non-fuzzy-based algorithms, such as SVM and MLP, showed a decreasing trend in RMSE, while the fuzzy-based algorithms show a neutral behavior to increasing iterations. In addition, GA-ANFIS and PSO-ANFIS algorithms presented a decreasing trend of RMSE after many iterations. However, it is noteworthy that the ANFIS algorithm kept the RMSE at 2.02 during all iterations, while the Cascaded-ANFIS algorithm gives the best RMSE. The Cascaded-ANFIS algorithm trains several FIS modules at a single iteration. Therefore, it is clear that the Cascaded ANFIS reaches a lower RMSE value in fewer iterations. Hence, this proves that the Cascaded-ANFIS algorithm performance saturates at the minimum number of iterations.

### 4.3. Confusion Matrix Analysis

The performance comparison of the modified Cascaded-ANFIS structure was evaluated using learning curves and the analysis of the confusion matrix. The overall confusion matrix was generated for all 131 classes when using the Cascaded-ANFIS algorithm for the classification.

Due to the unbalance samples in each class of the Fruit-360 dataset, the confusion matrix shows different colors at the top predictions. Therefore, summarized class information is given in Table 1 for further clarification.

**Figure 5:**
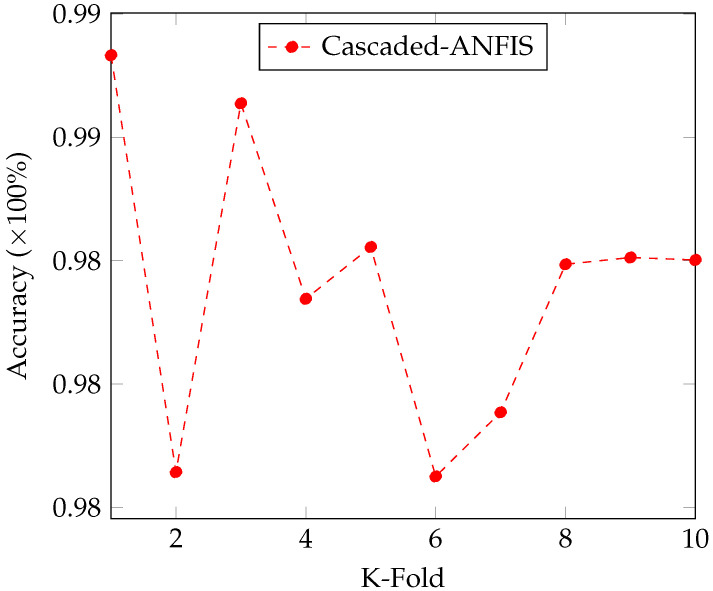
10-Fold Cross-Validation of the Accuracy of the Classifications.

Moreover, 10-fold cross validation was carried out to investigate the stability and robustness of the proposed algorithm. Figure 5 shows the resulting plot of the 10-fold cross validation. As shown in the figure, the accuracy remains between 98% and 99%. However, the average accuracy is calculated as 98.36%.

#### The Accuracy Evaluation of the Confusion Matrix

**Figure 6:**
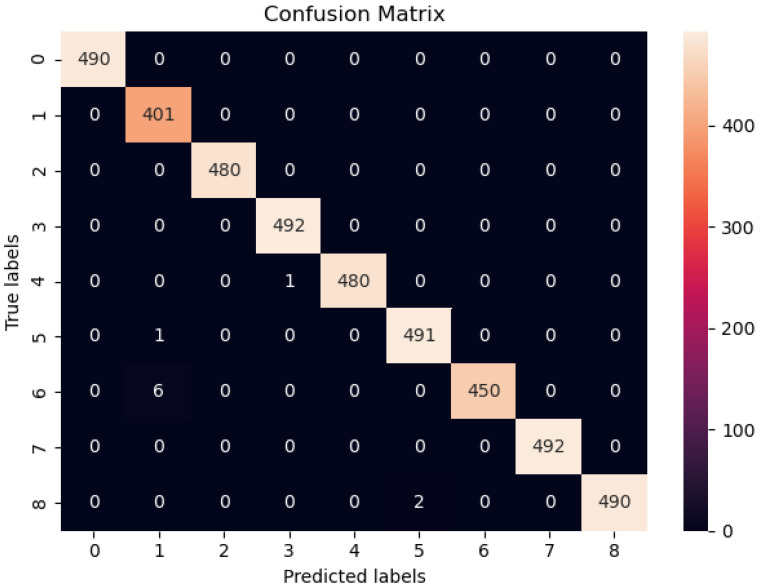
Confusion matrix for eight classes classification.

The accuracy of the class prediction was tested as a percentage of correctly predicted vs total tested images. A classification accuracy of 98.41% was achieved from the developed Cascaded-ANFIS model. In addition, the accuracy was checked for the confusion matrix using Equations (6)–(12). The dataset class samples were not balanced in Fruit-360, thus, the confusion matrix was generated for all 131 classes. Figure 6 presents a sample confusion matrix of eight classes. Table 2 shows the performance values for each of the parameters of the Cascaded-ANFIS algorithm-based classifier.

Four main parameters can be extracted from a confusion matrix such as True Positive (TP), False Positive (FP), True Negative (TN), and False Negative (FN). TP is the value of correct predictions of positives out of actual positive samples whereas the FP is the false positive representations of actual negative samples. TN is the accurate pessimistic predictions of actual negative samples, and FN is the false-negative samples. When the classes are unbalanced, the recall score is a good indicator of prediction success. It is the proportion of TP to a genuinely positive and FN in mathematics.

As can be clearly seen in Table 3, all the parameters are above the level of 0.98. This concludes that the classification performance of the Cascaded-ANFIS model is excellent and served well for the Fruit-360 dataset.

### 4.4. Comparison of Classification Accuracy against State-of-the-Art Algorithms

The literature showcases several attempts in classifying the Fruit-360 dataset at different years. The dataset is upgraded year by year, thus the usage of classes differs from study to study. Table 4 shows the best attempts in the past using different algorithms.

Ten different algorithms used to classify Fruit-360 data into its classes are summarized in Table 5. Importantly, all these attempts have been made during 2019 and 2020, and it is worth noting that these algorithms are based on CNN, such as CNN with Stochastic Gradient Descent with Momentum, CNN with Adaptive Moment Estimation, and Customized Inception V3.

The accuracy was measured as a percentage for all cases. The best results were found for the CNN approach when employing a Customized VGG16 network [21] and that was 99.27%. Though the results are higher in that research than in the proposed method in this study, the amount of data in the Fruit-360 dataset was lesser for Siddiqi [21]. Only used 72 classes with 48,249 samples were used by Siddiqi, while 131 classes with 90,380 samples were utilized for the presented study here.

In addition, the attempts presented in the year 2020 have noticeable accuracy reduction of 4–5% (96.25% [22] and 95% [23]). This could be due to the growth of the dataset. Therefore, the Cascaded-ANFIS model with a larger dataset has advantages in classification accuracy.

Usually, an expensive GPU is needed to run CNN-based algorithms due to high computational cost, whereas a conventional computer without GPU is enough for fuzzy-based ANFIS algorithms. Our experiments showed that the Cascaded-ANFIS algorithm could be implemented successfully using a computer without GPU, as shown in Table 3.

Moreover, CNN-based methods use an inbuilt feature extraction method, and the classification processes are performed using a fully connected neural network. However, the Cascaded-ANFIS study uses a fuzzy-based method and the feature extraction is performed outside of the leading classification algorithm. This characteristic of the algorithm allows the system to be modified using state-of-the-art feature extraction algorithms.

Furthermore, the Cascaded-ANFIS algorithm is a combination of multiple Fuzzy Inference Systems. Therefore, it can synthesize and infer good combinations automatically. Therefore, implementing the algorithm by distinct fuzzy reasoning methods can generate optimized solutions. The CNN-based processes operate as a black box, and the alternations of the functions can be challenging. Therefore, the Cascaded-ANFIS algorithm has many merits over the traditional CNNs.

## 5. Conclusions

The Fruit-360 dataset has 131 fruit classes with 90,483 sample images, and many researchers tried to classify fruits in the dataset using artificial intelligence and machine learning techniques. However, none of the previous attempts focused on handling all 131 fruit classes with a total number of fruit images. Therefore, a novel and successful attempt is presented in this research work in identifying all images in the Fruit-360 dataset using a Cascaded-ANFIS algorithm. The capability in image-based classification performance of the Cascaded-ANFIS algorithm was tested using nine feature descriptors. Thus, a robust and comprehensive Cascaded-ANFIS algorithm is presented in this research work.

The performance of the tested algorithm was tested using the learning curve and the confusion matrix. It can be concluded herein that the Cascaded-ANFIS algorithm outperformed all other state-of-the-art algorithms available for the specific task. The weighted precision, weighted recall, and weighted FScore reached their highest accuracies at 0.9843, 0.9841, and 0.9840, respectively for the unbalanced Fruit-360 dataset. Therefore, the results provide compelling evidence that the Cascaded-ANFIS algorithm can handle multiple class image classification problems with higher cost-effectiveness and comparative accuracy than the CNN-based methods in the past studies.

In addition, the algorithm showcased its capacities and capabilities in handling the total Fruit-360 data set at lower computational power. According to the results, it can be concluded that the Cascaded-ANFIS-based classifiers are suitable for real-time and cost-effective system implementations. The Cascaded-ANFIS architecture is an automatic cascade connection to the truth space approach of FIS. Therefore, Cascaded-ANFIS can rinse off the approximate reasoning part and make the reasoning of primary elements. Moreover, interaction selection of Cascaded-ANFIS works as the best choice with FIS. A significant limitation of using the Cascaded-ANFIS algorithm is that it may need a different structure to obtain better accuracy for each dataset, such as a specific number of levels and a total number of inputs. Therefore, future works should implement a generic Cascaded-ANFIS structure for image-based classification problems.

## Figures and Tables

**Table 1:** Model training performance with iterations.

No of Iterations	SVM	MLP	ANFIS	PSO-ANFIS	GA-ANFIS	Cascaded-ANFIS
1	1.98	3.28	2.02	1.91	1.92	0.31
10	1.61	0.95	2.02	1.91	1.92	0.24
100	1.20	0.43	2.02	1.43	1.83	0.20

**Table 2:** Sample distribution of the Fruit-360 dataset among some of the classes.

Class ID	Class Label	Number of Samples
0	Apple Braedurn	492
12	Apple Red Yellow 2	672
25	Cauliflower	702
32	Chestnut	450
42	Ginger Root	297
44	Grape Blue	984
66	Mangostan	300
73	Nut Pecan	534

**Table 3:** Performance of Confusion Parameters.

Metric	Performance Value
Average Accuracy	0.9841
Precision${}_{\mu }$	0.9841
Recall${}_{\mu }$	0.9841
FScore${}_{\mu }$	0.9841
Precision${}_{M}$	0.9846
Recall${}_{M}$	0.9849
FScore${}_{M}$	0.9845
Precision${}_{W}$	0.9843
Recall${}_{W}$	0.9841
FScore${}_{W}$	0.9840

**Table 4:** Configuration of the host computer.

Processor	Intel(R) Core(TM) i9-10900K CPU @ 3.70 GHz 3.70 GHz
Installed RAM	64.0 GB (63.9 GB usable)
Windows Edition	Windows 10 Education
HDD	4 TB
SSD	1 TB

**Table 5:** Comparison of classification accuracy against similar research work.

Reference Study	Algorithm	Size of the Dataset		Test Accuracy
		# Classes	# Samples	
Seda Postalcioglu (2019) [24]	CNN with Stochastic Gradient Descent with Momentum	48	50,590	98.08
	CNN with Adaptive Moment Estimation			98.83
	CNN with Root Mean Square Propagation			99.02
Raheel Siddiqi (2019) [21]	Customized Inception v3	72	48,249	99.1
	Customized VGG16			99.27
Ziliang Huang et al. (2019) [25]	Customized MobileNet	81	55,244	98.06
	Vanilla MobileNet			95.98
Sourodip Ghosh et al. (2020) [23]	ShufeNet V2	31	29,347	96.24
Ghazanfar Latif et al. (2020) [22]	DCNN	18	22,341	95
Jorg Martinet al. (2019) [49]	ResNet18	116	58,428	98.7
This Study (2022)	Cascaded-ANFIS	131	67,692	98.36

## Data Availability

The image data used to support the findings of this study have been deposited under the name of Fruit 360 in the Kaggle repository [47].

## Abbreviations

The following abbreviations are used in this manuscript:

MDPI	Multidisciplinary Digital Publishing Institute
ANFIS	Adaptive Network Based Fuzzy Inference System
RNN	Recurrent Neural Network
ANN	Artificial Neural Network
ARIMA	Auto Regressive Integrated Moving Average
FIS	Fuzzy Inference System
FL	Fuzzy Logic
ML	Machine Learning
PSO	Particle Swarm Optimization
GA	Genetic Algorithms
RMSE	Root Mean Square Error
CNN	Convolution Neural Network
ICA	Independent Component Analysis
PCA	Principle Component Analysis
MDS	Multi Dimensional Scaling
